# Energy Drink-Associated Mixed Liver Injury With Cholestatic Histology After Long-Term High-Volume Consumption: A Case Report

**DOI:** 10.7759/cureus.114422

**Published:** 2026-08-12

**Authors:** Monika Lis, Hubert Piwar, Maciej Drwęcki, Samuel Stróż, Krzysztof Pol

**Affiliations:** 1 Medicine, Mazovian "Bródnowski" Hospital, Warsaw, POL; 2 General Practice, Dr. Anna Gostynska Wolski Hospital, Warsaw, POL; 3 Internal Medicine and Gastroenterology, Mazovian "Bródnowski" Hospital, Warsaw, POL

**Keywords:** cholestasis, energy drinks, hepatotoxicity, liver biopsy, niacin

## Abstract

Energy drinks are multi-ingredient beverages whose clinically apparent hepatotoxicity is described mainly in case reports. A 25-year-old man with obesity and approximately three years of daily high-volume energy drink consumption (1-2.5 L/day) presented with epigastric pain, dark urine, jaundice, and systemic inflammation. His admission total bilirubin was 8.0 mg/dL, alanine aminotransferase 380 U/L, aspartate aminotransferase 187 U/L, gamma-glutamyl transferase 471 U/L, and alkaline phosphatase 232 U/L. The resulting R ratio was 4.5, indicating a mixed biochemical pattern. Computed tomography and magnetic resonance cholangiopancreatography excluded biliary obstruction and demonstrated hepatosplenomegaly, diffuse hepatic edema, and enlarged porta hepatis lymph nodes. Viral and autoimmune investigations were unrevealing. Core liver biopsy showed centrilobular and midzonal cholestasis with bilirubinostasis, minimal lobular inflammation, portal lymphogranulocytic infiltrates, preserved bile ducts, and no fibrosis. The energy drink exposure was stopped. Empiric antibiotics, ursodeoxycholic acid, and intravenous methylprednisolone were administered. By discharge on hospital day 18, bilirubin and C-reactive protein had substantially decreased; aminotransferases remained elevated but were declining from their peak. At four months, aspartate aminotransferase, alkaline phosphatase, and C-reactive protein were within the supplied institutional reference ranges, while alanine aminotransferase and gamma-glutamyl transferase had markedly improved. A retrospective updated Roussel Uclaf Causality Assessment Method score of 3 supported only a possible product-level association. This case illustrates that biochemical classification and histology may diverge and that causality should not be assigned to a single energy drink ingredient when the exact formulation, dose, and untreated dechallenge are unavailable.

## Introduction

Acute liver injury is recognized by a new elevation of serum aminotransferases and/or alkaline phosphatase (ALP), with or without hyperbilirubinemia, after the abnormality is confirmed and its severity is assessed. In suspected drug-induced liver injury (DILI), clinically significant injury is commonly defined by aspartate aminotransferase (AST) or alanine aminotransferase (ALT) levels greater than five times the upper limit of normal, ALP greater than twice the upper limit of normal, total bilirubin greater than 2.5 mg/dL with elevated liver enzymes, or an international normalized ratio (INR) greater than 1.5 with elevated liver enzymes [1]. The R ratio is calculated as (ALT/upper limit of normal)/(ALP/upper limit of normal): hepatocellular injury is defined by an R ratio ≥ 5, cholestatic injury by an R ratio ≤ 2, and mixed injury by an R ratio > 2 and <5 [1,2]. INR, mental status, glucose, and serial biochemical trends help determine severity. New coagulopathy (INR ≥ 1.5) and hepatic encephalopathy in a patient without pre-existing cirrhosis indicate acute liver failure and require urgent specialist assessment [1,2].

Clinical presentation ranges from asymptomatic biochemical abnormalities to fatigue, anorexia, nausea, vomiting, right upper quadrant or epigastric pain, pruritus, dark urine, pale stools, and jaundice; confusion or somnolence may indicate hepatic encephalopathy. Among symptomatic, clinically apparent cases, jaundice is a common and clinically important presentation. Common causes include acute viral hepatitis, drug- or supplement-induced injury, acetaminophen toxicity, alcohol-associated hepatitis, ischemic or hypoxic injury, autoimmune hepatitis, and acute biliary obstruction. Commonly implicated DILI exposure groups include antimicrobials, central nervous system agents such as antiepileptics, nonsteroidal anti-inflammatory drugs, and herbal and dietary supplements; acetaminophen is a major cause of dose-dependent intrinsic DILI [1,2]. Age and clinical context may additionally prompt evaluation for Wilson disease, vascular disorders, and less common infectious or metabolic causes. Diagnosis therefore integrates a detailed history of prescription, over-the-counter, supplement, alcohol, and toxin exposures with examination, serial liver biochemistry, tests of synthetic function, targeted virologic, autoimmune, and metabolic studies, and hepatobiliary imaging [1,2].

Energy drinks combine caffeine with variable amounts of sugars, B-group vitamins, amino acids, botanical extracts, and other compounds. In a survey of 75 products, caffeine was universal, whereas ingredient number and dose varied considerably; niacin was present in approximately two-thirds of products [3]. High caffeine exposure and niacin have both been discussed as possible contributors to adverse events associated with energy drinks [3-5]. However, available product-level reports do not establish caffeine as a specific cause of DILI, whereas dose- and formulation-dependent hepatotoxicity is better established for pharmacologic niacin, as discussed below. This compositional heterogeneity makes adverse-event attribution difficult. Energy drink use has been associated with cardiovascular, neurologic, metabolic, renal, and gastrointestinal effects, but clinically apparent liver injury remains uncommon and is supported largely by individual reports rather than controlled evidence [4,5].

DILI is a diagnosis of exclusion. The initial biochemical pattern is classified using the R ratio, while imaging, competing-cause testing, exposure timing, dechallenge, and, when informative, histology contribute to causal assessment [1,2]. Biochemical and histologic phenotypes need not coincide. We report a young man with prolonged high-volume energy drink exposure, a mixed biochemical pattern, and predominantly cholestatic histology. The case is presented as an association with a multi-ingredient beverage rather than proof of toxicity from any one constituent.

## Case presentation

A 25-year-old male manual worker with obesity presented in April 2024. One day before admission, he developed sudden severe epigastric pain shortly after consuming an energy drink. Marked weakness, dark urine, and jaundice were present by the time of admission. He denied vomiting, diarrhea, pale stools, weight loss, previous similar episodes, known chronic liver disease, surgery, or drug allergies. Palpitations, presyncope, and syncope were not documented in the supplied record. He used electronic cigarettes. He denied recreational drug use and habitual alcohol misuse; his only reported recent alcohol exposure was 500 mL of beer three days before presentation. His mother had type 2 diabetes mellitus and hypertension, and his father had psoriasis. No pre-illness liver biochemistry or previous health-examination results were available in the supplied record.

The patient reported consuming energy drinks daily for approximately three years, usually 1-2.5 L/day. The retrospective record named a commercially available brand, but the exact product variant, can size, formulation, number of servings, lot, and ingredient label were not preserved. No recent dose escalation or switch in product was documented. Consequently, the daily caffeine, niacin, and other ingredient doses could not be reconstructed reliably.

At emergency department presentation, blood pressure was 136/89 mmHg, heart rate 114 beats/minute, respiratory rate 18 breaths/minute, oxygen saturation 98% on room air, and temperature 36.8°C. He was visibly jaundiced and had epigastric tenderness without documented peritoneal signs. Body weight was 100 kg, and height was 171 cm, corresponding to a body mass index of 34.2 kg/m². Initial laboratory testing demonstrated leukocytosis and a mixed liver test abnormality (Table 1). The source laboratory system showed total bilirubin 8.0 mg/dL, ALT 380 U/L, AST 187 U/L, gamma-glutamyl transferase (GGT) 471 U/L, and ALP 232 U/L. Using the institutional upper limits of normal of 55 U/L for ALT and 150 U/L for ALP, the R ratio was 4.5, indicating mixed injury.

**Table 1:** Selected laboratory results during hospitalization and follow-up Liver test and C-reactive protein values and their reference limits are transcribed from screenshots of the source laboratory system, which were treated as the primary source. Other values were transcribed from the supplied clinical summary. For high-density lipoprotein (HDL) cholesterol, ≥40 mg/dL is shown as a commonly used desirable clinical cutoff for men rather than an institutional laboratory reference range. A dash indicates that no value was available in the supplied materials. No pre-illness lipid profile, repeat lipid profile after recovery, or triglyceride result was available. ALT: alanine aminotransferase; ALP: alkaline phosphatase; AST: aspartate aminotransferase; GGT: gamma-glutamyl transferase

Parameter (reference range or clinical cutoff)	Hospital day 1	Hospital day 3	Hospital day 18	Four-month follow-up
White blood cells (4.0-10.0 × 10³/µL)	13.77	9.37	13.53	9.16
Neutrophils (1.8-7.7 × 10³/µL)	11.18	7.80	9.73	5.24
Hemoglobin (14.0-18.0 g/dL)	-	13.3	14.5	16.3
Platelets (150-400 × 10³/µL)	-	171	289	272
AST (0-35 U/L)	187	94	201	32
ALT (0-55 U/L)	380	226	1,007	73
GGT (0-66 U/L)	471	324	386	172
ALP (40-150 U/L)	232	222	297	132
Total bilirubin (0.3-1.2 mg/dL)	8.00	5.19	2.75	-
C-reactive protein (0.0-5.0 mg/L)	119.8	147.6	5.4	2.3
Procalcitonin (<0.5 ng/mL)	0.43	0.70	-	-
Total cholesterol (<190 mg/dL)	-	-	610	-
High-density lipoprotein cholesterol (desirable ≥40 mg/dL in men)	-	-	26	-
Low-density lipoprotein cholesterol (<115 mg/dL)	-	-	459	-

**Figure 1:**
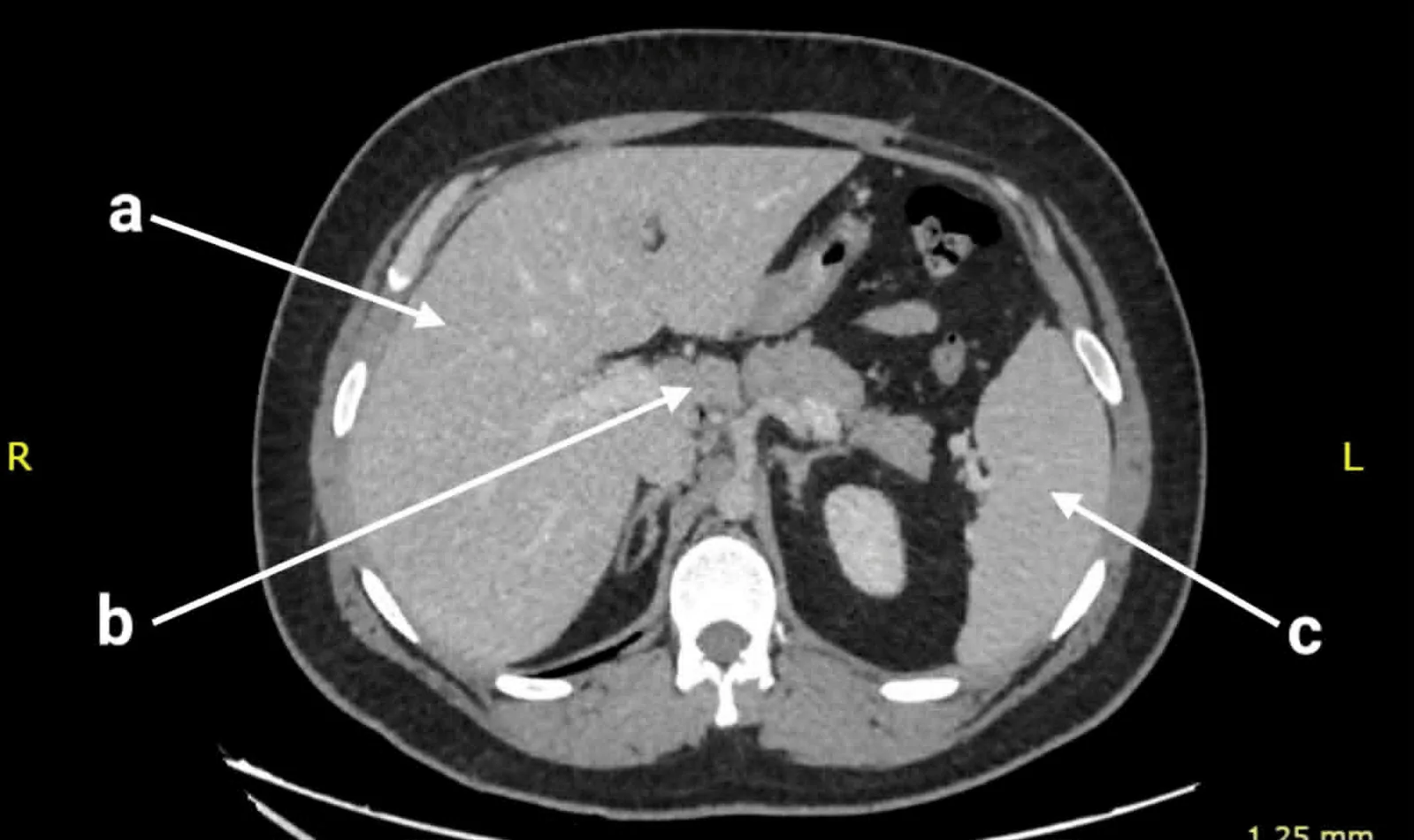
Contrast-enhanced computed tomography of the upper abdomen Axial contrast-enhanced computed tomography demonstrates (a) hepatomegaly with mildly heterogeneous hepatic enhancement, (b) enlarged lymph nodes at the porta hepatis, and (c) splenomegaly. The intrahepatic bile ducts are not dilated.

Abdominal ultrasonography showed a borderline enlarged liver with mild steatotic echogenicity, a 5 × 9 mm hypoechoic focus in segment III, a contracted gallbladder with a 4 mm wall, and splenomegaly. Contrast-enhanced computed tomography demonstrated mild hepatomegaly with heterogeneous arterial parenchymal enhancement, splenomegaly to approximately 15 cm, a common hepatic nodal conglomerate measuring approximately 35 × 15 mm, and an enlarged hepatoduodenal ligament node measuring 18 × 15 mm. No calcified gallstones or biliary duct dilation were seen (Figure 1).

On hospital day 3, the patient developed a fever of 38.0°C. Blood cultures were obtained after antimicrobial treatment had begun and showed no aerobic or anaerobic growth. C-reactive protein (CRP) rose to 147.6 mg/L, and procalcitonin was 0.70 ng/mL. Initial empiric ceftriaxone plus metronidazole was later broadened to piperacillin/tazobactam because of persistent systemic inflammation and concern for an occult biliary or other bacterial infection. The supplied records contain one inconsistent narrative entry naming ciprofloxacin rather than ceftriaxone as the initial agent; the discharge medication chronology was treated as the primary source for this report.

On hospital day 4, cough, pleuritic right infrascapular pain, sinus tachycardia, transient hypotension to approximately 90/75 mmHg, and oxygen desaturation to approximately 90% developed. Computed tomography pulmonary angiography excluded pulmonary embolism and showed mild dependent atelectatic or hypoventilatory change and subcarinal lymph nodes measuring approximately 12-13 mm. A single therapeutic dose of enoxaparin had been given while pulmonary embolism was being evaluated. Tests for SARS-CoV-2 and influenza were negative.

**Figure 2:**
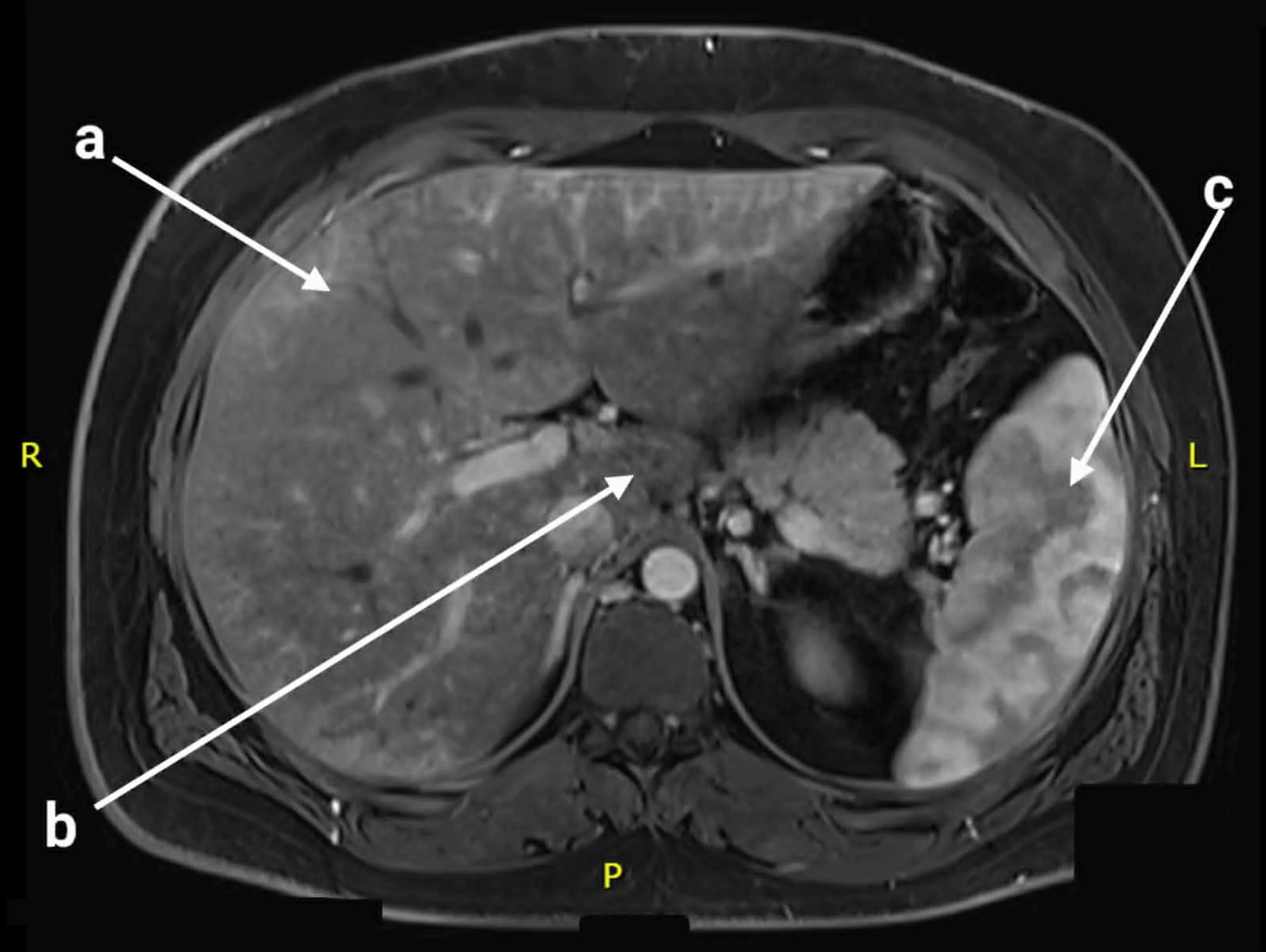
Contrast-enhanced magnetic resonance imaging of the upper abdomen Axial contrast-enhanced magnetic resonance imaging demonstrates (a) an enlarged, edematous liver, (b) enlarged porta hepatis lymph nodes, and (c) splenomegaly. Magnetic resonance cholangiopancreatography showed no biliary duct dilation or choledocholithiasis.

Magnetic resonance imaging (MRI) with magnetic resonance cholangiopancreatography (MRCP) showed hepatomegaly to 17.5 cm with diffuse parenchymal edema, no focal liver lesion, and no convincing steatosis. The gallbladder contained no visible stones; its wall was mildly edematous, with a small amount of pericholecystic fluid. The intrahepatic and extrahepatic ducts were not dilated and contained no visible stones. The spleen measured approximately 17 cm. Two common hepatic nodes formed a 25 × 16 mm conglomerate, and a hepatoduodenal ligament node measured 19 × 15 mm. The abdominal aorta was patent and nondilated. Small bilateral pleural effusions were present (Figure 2).

Subsequent abdominal ultrasonography showed normal hepatic echogenicity, a nondilated portal vein with preserved hepatopetal flow, and patent hepatic veins. The intrahepatic ducts and common bile duct remained nondilated, and no ascites was seen.

Serologic evaluation was reported as negative for acute hepatitis A, B, C, D, and E. Antinuclear, antimitochondrial, anti-smooth muscle, anti-liver-kidney microsomal, anti-soluble liver antigen, and perinuclear antineutrophil cytoplasmic antibodies were negative. Immunoglobulin G was 941 mg/dL, and immunoglobulin M was 70 mg/dL. Cytomegalovirus and Epstein-Barr virus testing was consistent with past rather than acute infection. No obstructive process was identified by ultrasonography, computed tomography, or MRCP. Routine serum ethanol testing was reported as negative. INR, albumin, acetaminophen concentration, a broad serum or urine toxicology screen, measured serum osmolality with a calculated osmolal gap, targeted assays for methanol, ethylene glycol, and isopropanol, ceruloplasmin, and alpha-1 antitrypsin results were not available in the supplied source material.

An ultrasound-guided core liver biopsy was performed on hospital day 6. The 11 mm core contained 11 portal tracts. Histology showed cholestasis and bilirubinostasis in hepatocytes around central veins and in midzonal areas, minimal lobular inflammatory activity, and moderate-to-dense portal lymphocytic and granulocytic infiltrates. Bile ducts were preserved without destruction, although their walls were inflamed. There was no evidence of a lymphoproliferative process. Chromotrope 2R-aniline blue and reticulin stains showed no fibrosis, cytokeratin 7 showed no ductular reaction, rhodanine staining was negative for copper deposition, and periodic acid-Schiff with diastase showed no cytoplasmic inclusions. The pathologist concluded that the findings represented cholestatic liver injury and that a toxic effect from an exogenous substance could not be excluded.

The hospital course and principal decisions are summarized in Table 2. In addition to stopping energy drinks, management included intravenous fluids, vitamin K, empiric antimicrobials, and symptom-directed supportive care. Ursodeoxycholic acid and intravenous methylprednisolone 60 mg/day were started on hospital day 10 after the preliminary biopsy interpretation suggested cholestatic injury. The rationale for corticosteroids was the severity and ongoing biochemical progression despite exclusion of major viral, autoimmune, and obstructive causes; no hypersensitivity syndrome was documented.

**Table 2:** Clinical timeline CRP: C-reactive protein; MRI: magnetic resonance imaging; MRCP: magnetic resonance cholangiopancreatography; AST: aspartate aminotransferase; ALP: alkaline phosphatase; GGT: gamma-glutamyl transferase; ALT: alanine aminotransferase

Time	Event
Day 0	Severe epigastric pain after an energy drink; weakness, dark urine, and jaundice were present by admission
Day 1	Hospital admission; mixed biochemical liver injury, systemic inflammation, hepatosplenomegaly, and porta hepatis lymphadenopathy identified
Day 3	Fever; cultures obtained after antibiotics; CRP increased to 147.6 mg/L
Day 4	Transient hypoxemia, tachycardia, and hypotension; pulmonary embolism excluded; MRI/MRCP confirmed diffuse hepatic edema without biliary obstruction
Day 5	Antimicrobial therapy broadened because of persistent inflammatory markers and concern for occult infection
Day 6	Ultrasound-guided core liver biopsy
Day 9	Esophagogastroduodenoscopy normal; echocardiography showed preserved left ventricular ejection fraction
Day 10	Ursodeoxycholic acid and intravenous methylprednisolone started after preliminary biopsy findings
Day 18	Discharged in good clinical condition; bilirubin and CRP markedly lower, with aminotransferases declining from their peak
Four months	AST, ALP, and CRP within supplied reference ranges; ALT and GGT substantially improved but GGT remained elevated

The biochemical course was not uniformly improving. By hospital day 3, AST, ALT, and total bilirubin had initially decreased to 94 U/L, 226 U/L, and 5.19 mg/dL, respectively, while CRP had increased to 147.6 mg/L. ALT subsequently peaked at approximately 1,475 U/L during hospitalization and had decreased to 1,007 U/L by discharge on hospital day 18. At discharge, the patient was in good general condition, and his vital signs had normalized; the exact day of symptom resolution was not documented. AST was 201 U/L, GGT 386 U/L, ALP 297 U/L, total bilirubin 2.75 mg/dL, and CRP 5.4 mg/L at discharge. At laboratory follow-up four months later, AST was 32 U/L, ALT 73 U/L, GGT 172 U/L, ALP 132 U/L, and CRP 2.3 mg/L. A bilirubin value and a detailed symptom assessment were not available for that visit. Rechallenge with an energy drink was neither performed nor recommended.

## Discussion

This case combines prolonged high-volume exposure to a multi-ingredient beverage, a mixed biochemical liver injury pattern, cholestatic histology, and substantial improvement after exposure cessation and treatment. The R ratio was 4.5, while biopsy emphasized centrilobular and midzonal cholestasis and bilirubinostasis. Such discordance is plausible: biochemical categories are useful clinical descriptors but do not reliably predict the dominant histologic pattern [6,7]. In a series of suspected DILI biopsies, biochemical hepatocellular and cholestatic classifications did not show consistent correspondence with the respective histologic patterns [6]. Histology in this case was useful chiefly to characterize injury, document the absence of fibrosis and bile duct loss, and exclude infiltrative or lymphoproliferative disease; it did not identify a specific causative compound [1,8].

Published energy drink-associated liver injury spans mild enzyme elevations, severe acute hepatitis, acute liver failure, and transplantation (Table 3) [9-15]. Reported exposure patterns vary from short binges to months of daily use. Several reports contain important competing factors, including a transplanted liver, binge alcohol use, hepatitis C viremia, diabetes with fatty liver disease, malignancy, cannabis exposure, and uncertainty about the product or amount [10-15]. The present case differs in its approximately three-year exposure and cholestatic-predominant histology, but that long latency also weakens a simple temporal inference. No recent change in volume or formulation was documented.

**Table 3:** Selected published reports of energy drink-associated liver injury

Report	Exposure	Main phenotype and relevant competing factors	Outcome
Vivekanandarajah et al. [9]	22-year-old woman; 10 cans/day for two weeks	Severe hepatocellular injury; exact culprit ingredient unproven	Biochemical resolution after cessation
Apestegui et al. [10]	16-year-old male liver recipient; two temporally related binges	Recurrent hepatitis in a transplanted liver; positive unintentional re-exposure	Recovery after cessation
Huang et al. [11]	36-year-old man; three sugar-free drinks/day for one year	Acute liver failure; long-standing binge alcohol exposure	Liver transplantation
Harb et al. [12]	50-year-old man; four to five drinks/day for three weeks	Severe acute hepatitis with cholestasis; low-level hepatitis C viremia	Recovery with supportive care
Uwaifo [13]	46-year-old man; two to three 16 oz cans/day for four months	Mild hepatitis with gastritis and pancreatitis; diabetes and fatty liver disease	Improvement after cessation
Al Yacoub et al. [14]	62-year-old woman; five to six 16 oz cans/day	Acute hepatitis and kidney injury; advanced malignancy and limited intake	Recovery with supportive care
Jesse et al. [15]	21-year-old man; uncertain multi-brand binge during >30-hour gaming event	Acute liver failure; uncertain dose and cannabis exposure	Liver transplantation
Present case	25-year-old man; 1-2.5 L/day for approximately three years	Mixed biochemical injury with cholestatic histology; obesity and systemic inflammation	Marked biochemical improvement by four months

Niacin has frequently been proposed as a candidate hepatotoxin in energy drink reports. Pharmacologic niacin, particularly certain sustained-release preparations, can cause hepatocellular or cholestatic injury, usually at gram-level doses used for lipid modification [16,17]. A recent report described cholestatic DILI during 2,500 mg/day of extended-release niacin [18]. By contrast, the niacin content of commercially marketed energy drinks is generally much lower, although it can exceed daily nutritional requirements and varies across products [3]. In the present case, the exact product formulation and serving count were unavailable. Niacin or nicotinamide exposure therefore could not be quantified, and neither niacin nor any other individual ingredient can be designated as the causative agent.

The competing explanation of inflammation- or infection-associated cholestasis deserves particular emphasis. The patient had fever, CRP values of 119.8-147.6 mg/L, transient tachycardia and hypotension, porta hepatis and subcarinal lymphadenopathy, splenomegaly, and small pleural effusions. No source was established, and blood cultures were negative, but they were drawn after antibiotics. These features prevent confident exclusion of an occult infectious or systemic inflammatory process. Conversely, the liver injury and jaundice were already present before antimicrobial exposure, so ceftriaxone, metronidazole, piperacillin/tazobactam, or other hospital medications cannot explain the onset, although they may have influenced the later aminotransferase rise.

The retrospective updated Roussel Uclaf Causality Assessment Method (RUCAM) score was 3, corresponding to a possible association (Table 4) [19]. The assessment was applied to the consumed energy drink product as a whole. A component-level score would be invalid because the exact formulation and dose were unknown. The treated dechallenge was deliberately scored as unavailable: corticosteroids and ursodeoxycholic acid may alter the natural biochemical course, and the updated RUCAM guidance advises against crediting such a course as spontaneous dechallenge [19]. The lack of re-exposure, long latency, unresolved inflammatory alternative, and absence of an untreated course appropriately limit certainty.

**Table 4:** Retrospective updated RUCAM (Roussel Uclaf Causality Assessment Method) assessment for the energy drink product This is an author-created, case-specific application of the published updated RUCAM framework [19]; no published table was reproduced.

Updated RUCAM item for mixed/cholestatic injury	Score	Case-specific rationale
Time to onset	+1	Exposure exceeded 90 days before onset
Course after cessation	0	Steroid and ursodeoxycholic acid treatment precluded assessment of untreated dechallenge
Risk factors	0	Age < 55 years; no qualifying alcohol use documented
Concomitant exposure	0	No pre-event concomitant drug was documented as a plausible cause
Search for alternative causes	+1	Major viral, autoimmune, and obstructive causes were investigated, but systemic inflammation remained an unresolved alternative
Previous hepatotoxicity information	+1	Published case reports describe liver injury associated with energy drink products
Unintentional re-exposure	0	No re-exposure occurred
Total	3	Possible association

Stopping the suspected product and supportive care are the foundations of DILI management [1,2]. Evidence supporting corticosteroids outside selected immune-mediated presentations is limited, and ursodeoxycholic acid has not been established as disease-modifying therapy for DILI, although it is commonly used and is generally well tolerated [1]. Therefore, the biochemical improvement after methylprednisolone and ursodeoxycholic acid should not be interpreted as confirmation of either mechanism or causality. His obesity, mild steatotic echogenicity on admission ultrasonography, and abnormal day-18 lipid profile raise the possibility of an underlying metabolic or steatotic background. However, MRI showed no convincing steatosis, subsequent ultrasonography showed normal hepatic echogenicity, the biopsy did not report steatosis or fibrosis, and no pre-illness liver tests or lipid profile were available. The residual ALT and GGT elevations at four months may represent incomplete recovery and do not independently establish metabolic dysfunction-associated steatotic liver disease. Obesity or steatotic liver disease may have influenced susceptibility, but evidence that either increases DILI risk is agent-specific and cannot be extrapolated directly to energy drinks [20].

This report has several limitations. Exposure was reconstructed retrospectively, without a retained can, label, lot number, or measured ingredient dose. No pre-illness liver biochemistry, health-examination results, or metabolic profile was available, precluding confirmation of normal baseline liver tests or a pre-existing chronic liver disorder. Although the patient denied acetaminophen use and habitual alcohol misuse and routine serum ethanol testing was negative, history and a single ethanol result cannot completely exclude an occult toxic exposure. No acetaminophen concentration, broad toxicology screen, measured serum osmolality with a calculated osmolal gap, or targeted methanol, ethylene glycol, and isopropanol assays were documented. An acetaminophen concentration would have strengthened the evaluation because exposure histories may be incomplete [1]. Targeted toxic alcohol testing would have been most informative if the exposure history, acid-base status, or osmolal gap suggested such poisoning. INR, albumin, ceruloplasmin, and alpha-1 antitrypsin results were also unavailable. The clinical course was modified by antibiotics, corticosteroids, and ursodeoxycholic acid, and the inflammatory syndrome was not fully explained. There was no re-exposure, product analysis, or detailed four-month symptom assessment. These limitations are why the case supports a possible product-level association, not proven ingredient-specific DILI.

## Conclusions

In this single patient, chronic high-volume energy drink exposure was temporally associated with mixed biochemical liver injury and cholestatic histology. The small and heterogeneous case report literature, possible metabolic or steatotic susceptibility, systemic inflammation, and a treatment-modified course preclude generalization and do not establish causality, incidence, or a dose-response relationship. The case nevertheless highlights the value of documenting the volume, duration, brand, and formulation of energy drink use when evaluating otherwise unexplained liver injury.

Biochemical classification and histologic phenotype may diverge, and a multi-ingredient beverage should not be reduced to a single presumed culprit when its formulation and dose are unknown. Careful exposure documentation, serial ALT, ALP, bilirubin, and INR measurements, structured causality assessment, and sufficiently detailed follow-up would make future reports more informative.
